# Improved Protein Structure Prediction Using a New Multi‐Scale Network and Homologous Templates

**DOI:** 10.1002/advs.202102592

**Published:** 2021-10-31

**Authors:** Hong Su, Wenkai Wang, Zongyang Du, Zhenling Peng, Shang‐Hua Gao, Ming‐Ming Cheng, Jianyi Yang

**Affiliations:** ^1^ School of Mathematical Sciences Nankai University Tianjin 300071 China; ^2^ Research Center for Mathematics and Interdisciplinary Sciences Shandong University Qingdao 266237 China; ^3^ College of Computer Science Nankai University Tianjin 300071 China

**Keywords:** deep learning, homology modeling, protein structure prediction

## Abstract

The accuracy of *de novo* protein structure prediction has been improved considerably in recent years, mostly due to the introduction of deep learning techniques. In this work, trRosettaX, an improved version of trRosetta for protein structure prediction is presented. The major improvement over trRosetta consists of two folds. The first is the application of a new multi‐scale network, i.e., Res2Net, for improved prediction of inter‐residue geometries, including distance and orientations. The second is an attention‐based module to exploit multiple homologous templates to increase the accuracy further. Compared with trRosetta, trRosettaX improves the contact precision by 6% and 8% on the free modeling targets of CASP13 and CASP14, respectively. A preliminary version of trRosettaX is ranked as one of the top server groups in CASP14's blind test. Additional benchmark test on 161 targets from CAMEO (between Jun and Sep 2020) shows that trRosettaX achieves an average TM‐score ≈0.8, outperforming the top groups in CAMEO. These data suggest the effectiveness of using the multi‐scale network and the benefit of incorporating homologous templates into the network. The trRosettaX algorithm is incorporated into the trRosetta server since Nov 2020. The web server, the training and inference codes are available at: https://yanglab.nankai.edu.cn/trRosetta/.

## Graphical abstract

trRosettaX is an improved version of trRosetta for protein structure prediction. The major improvement over trRosetta consists of two folds. The first is the application of a new multi‐scale network, i.e., Res2Net, for improved prediction of inter‐residue geometries. The second is an attention‐based module to exploit multiple homologous templates to increase the accuracy further.

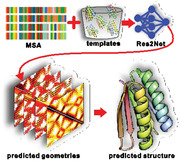

## 1 Introduction

Compared with template‐based modeling,^[^ ^1^, ^2^, ^3^, ^4^, ^5^ ^]^ *de novo* protein structure prediction is known to be slow and inaccurate for many years, mostly due to the difficulty in designing accurate force fields and efficient sampling algorithms.^[^ ^6^, ^7^, ^8^, ^9^, ^10^, ^11^, ^12^ ^]^ Significant efforts have been made to improve *de novo* protein structure prediction by introducing additional constraints, leading to remarkable progress in the last decade.

The progress can be divided into three stages broadly. The first stage is contact‐assisted folding with inter‐residue contacts predicted from multiple sequence alignment (MSA). The hypothesis is that two residues that are close in space (i.e., in contact) should have correlated patterns of mutations, which can be deduced from the MSA. In fact, inter‐residue contact prediction was first proposed in the 1990s by Göbel and colleagues.^[^ ^13^ ^]^ But its precision remained notoriously low for about 20 years,^[^ ^14^ ^]^ especially for hard targets that do not have homologous templates in the Protein Data Bank (PDB)^[^ ^15^ ^]^ (≈20% for the top *L*/5 long‐range (i.e., sequence separation ≥ 24) contacts; *L* is the length of a target). This is mostly due to the effect of indirect coupling caused by transmitting between residues.^[^ ^16^ ^]^ The development of the direct coupling analysis (DCA)^[^ ^17^ ^]^ and the exponential growth of protein sequence data boosted the contact precision significantly starting from around 2010^[^ ^18^ ^]^ (increase to about 30% for the top *L*/5 long‐range contacts). Representative methods include EVfold,^[^ ^19^ ^]^ PSICOV,^[^ ^20^ ^]^ plmDCA,^[^ ^21^ ^]^ GREMLIN,^[^ ^22^ ^]^ CCMpred,^[^ ^23^ ^]^ and so on. Many groups successfully demonstrate that the improved contacts can be used to fold protein structures that are not possible before.^[^ ^24^, ^25^, ^26^, ^27^ ^]^ A diverse set of model building protocols were used, including the Monte Carlo simulations in I‐TASSER,^[^ ^1^ ^]^ fragment assembly in Rosetta,^[^ ^6^ ^]^ Crystallography and NMR System (CNS)^[^ ^28^ ^]^ etc.

The second stage is distance‐assisted folding with inter‐residue distance predicted by deep learning. In 2016, the Xu group demonstrated that with the application of the residual network (ResNet),^[^ ^29^ ^]^ the precision of predicted contacts could be doubled compared with DCA‐based methods.^[^ ^30^ ^]^ This is mostly because the contact map is predicted globally in ResNet rather than separated as individual residue pairs. In addition, the combination of ResNet and LSTM^[^ ^31^ ^]^ was shown to yield more precise contact prediction than the pure ResNet.^[^ ^32^ ^]^ The contact prediction was later extended to distance prediction by Xu.^[^ ^33^ ^]^ DeepMind developed AlphaFold,^[^ ^34^ ^]^ the CASP13 winner, in which the predicted distance distribution was used to score structure models. trRosetta^[^ ^35^ ^]^ was developed on these advances by proposing inter‐residue orientations and efficient energy minimization‐based structure realization in Rosetta. Besides the methods mentioned above, there are many other deep learning‐based structure prediction methods, such as DMPfold,^[^ ^36^ ^]^ tFold,^[^ ^37^ ^]^ MULTICOM,^[^ ^38^ ^]^ CONFOLD,^[^ ^39^ ^]^ C‐I‐TASSER,^[^ ^40^ ^]^ CopulaNet,^[^ ^41^ ^]^ and so on.

The third stage is the end‐to‐end prediction with the attention and rotation‐equivariant networks. To the best of our knowledge, DeepMind's AlphaFold2^[^ ^42^ ^]^ is the first working method. Based on the proposed invariant point attention (IPA), a structure module is used to produce the 3D atom coordinates from single‐residue and residue‐pair representations, which are generated from MSA and structure templates using an attention‐based network (called Evoformer). As demonstrated in the CASP14 experiment, AlphaFold2 represents one of the breakthroughs in the field of protein structure prediction, in which about two thirds of the CASP14 targets were predicted with GDT‐TS score^[^ ^43^ ^]^ higher than 90. The success of AlphaFold2 proves that it is possible to consistently predict protein structure with high accuracy. The detailed description of the AlphaFold2 methodology and the release of its source codes will accelerate the development of the field. Though the high demand of computing resource for training becomes the bottleneck for most academic groups, we firmly believe that more new methods will be developed in future even with limited computing resource. In fact, the RoseTTAFold^[^ ^44^ ^]^ from the Baker group represents one of such examples.

In this work, we present trRosettaX, an improved version of trRosetta. The major improvement lies in two aspects: one is a new multi‐scale network and the other is the automated integration of homologous templates with an attention‐based module. A preliminary version of trRosettaX was ranked as one of the top server groups in the recent CASP14 experiment. Benchmark tests on the CASP13, CASP14, and the CAMEO datasets show that trRosettaX significantly outperforms trRosetta and is competitive with other state‐of‐the‐art automated methods.

## 2 Results and Discussions

### 2.1 Overview of trRosettaX

**Figure 1:**
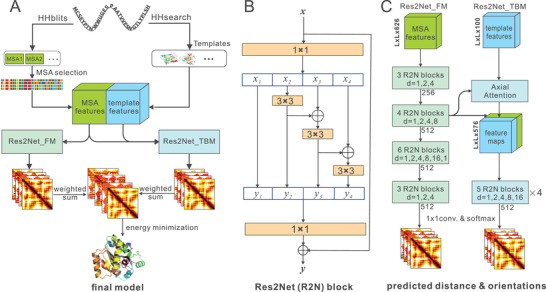
Predict inter‐residue geometries and protein 3D structure using MSA and homologous templates. A) Overview of trRosettaX. The proposed trRosettaX first predicts inter‐residue geometries based on two Res2Net‐based networks. The first network (Res2Net_FM) is for *de novo* prediction using features derived from the input MSA. The second network (Res2Net_TBM) includes additional features extracted from multiple homologous templates, combined by an attention‐based module, together with the MSA‐derived features. The final predictions are obtained by a pixel‐wise combination of the predictions from the both networks, which are then converted into distance and orientation constraints to guide the structure folding by energy minimization. B) A basic Res2Net block. (C) Two Res2Net‐based architectures to predict the inter‐residue geometries. Dilated convolutions are employed with different dilation rates (denoted by *d*).

As illustrated in **Figure** **1**A, trRosettaX is an improved version of trRosetta in two major aspects, i.e., an improved multi‐scale network and the inclusion of homologous templates. Similar to trRosetta, the new pipeline consists of two main steps: inter‐residue geometries (distance and orientations) prediction and restraint‐guided structure folding. Given a target protein sequence, multiple MSAs are generated and the optimal MSA is selected based on the probability of the top predicted contacts, similar to our previous works.^[^ ^35^, ^45^ ^]^ The geometry prediction is based on the new multi‐scale network Res2Net^[^ ^46^ ^]^ (see Experimental Section and Figure 1B), which is an improved version of the popular ResNet.^[^ ^29^ ^]^ Two Res2Net‐based networks (Figure 1C) are designed. The first network is for *de novo* prediction using features derived from the input MSA (denoted by Res2Net_FM; the corresponding modeling method is denoted by trRosettaX_FM). The second network (denoted by Res2Net_TBM) includes additional features extracted from homologous templates (detected by HHsearch^[^ ^47^ ^]^) together with the MSA‐derived features. The predicted geometries by both networks are combined in a pixel‐wise manner (see Experimental Section) to yield the final geometry prediction. The predicted geometries are then converted into distance and orientation constraints to guide the structure folding by energy minimization. trRosettaX combines the outputs from Res2Net_FM and Res2Net_TBM for structure modeling; while trRosettaX_FM only uses Res2Net_FM's output for structure modeling.

### 2.2 Benchmark Results

We first compare trRosettaX with trRosetta on the CASP13 and CASP14 datasets. The CASP13 dataset consists of 31 free‐modeling (FM) targets which was used in the trRosetta. The CASP14 dataset consists of 91 targets (we do not have experimental structures for the other five CASP14 targets), including 23 FM targets, 14 FM/template‐based modeling (FM/TBM) targets and 54 TBM targets. Corresponding to the two steps in trRosettaX, the evaluation consists of two aspects, i.e., predicted inter‐residue distance and structure models. The predicted distance distribution is first converted into binary contact by summing up the probabilities corresponding to the distance bins with distance ≤ 8 Å. Then the predicted contacts are ranked based on the probability and assessed using the metrics *precision*, which is defined as the number of true contacts divided by the number of predicted contacts. In general, the top *L*/*k* predicted contacts are assessed (*L* is the length of the query sequence). In addition, based on the separation of the *i‐*th and the *j‐*th residues along the sequence (i.e., |*i*‐*j*|), the contacts fall into three classes: short range [6, 12), medium range [12, 24), and long range [24, *∞*). The first class is skipped due to its relatively low importance to protein structural modeling. The precision of the top *L* predicted long‐ and medium‐range contacts (i.e., sequence separation ≥ 12) is used in the subsequent evaluation. The quality of a predicted structure model is measured by TM‐score^[^ ^48^ ^]^ with respect to the experimental structure. A TM‐score higher than 0.5 suggests that the model has a correct fold.^[^ ^49^ ^]^

#### 2.2.1 Res2Net Consistently Improves Inter‐Residue Distance Prediction over ResNet

Since first applied in RaptorX‐Contact,^[^ ^30^ ^]^ ResNet has been used by almost all existing deep‐learning‐based contact/distance prediction methods.^[^ ^34^, ^35^, ^36^ ^]^ In this work, instead of ResNet, the recently‐proposed multi‐scale network Res2Net is employed in trRosettaX. It was shown that Res2Net can extract features from input images more efficiently than ResNet due to its ability in multi‐scale representation (see Figure S3, Supporting Information).^[^ ^50^ ^]^

**Figure 2:**
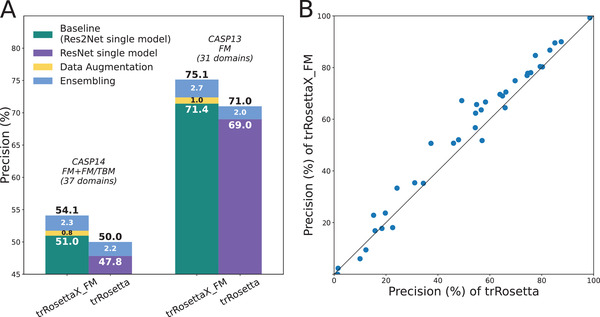
Precision of the top *L* predicted contacts (sequence separation ≥ 12) on targets from CASP13 and CASP14. A) Contribution of different factors to the improvement in trRosettaX_FM. B) A head‐to‐head comparison between trRosettaX_FM and trRosetta on CASP14's FM+FM/TBM targets. Note that the same set of MSAs are used in both trRosetta and trRosettaX_FM.

We assess the performance of predicted contacts on 37 targets from CASP14 (23 FM and 14 FM/TBM domains) and 31 CASP13 FM targets. **Figure** **2**A shows the precision of the top *L* predicted contacts (sequence separation ≥ 12) on both datasets. For the CASP14 targets (the left panel of Figure 2A), the baseline single‐Res2Net‐based model has a mean precision of 51%, which is 6.7% higher than the precision (47.8%) of the single‐ResNet‐based model. The precision is improved further from 51% to 51.8% by using the data augmentation in training (see Experimental Section and Figure S2, Supporting Information). A mean‐ensemble‐based Res2Net model achieves a precision of 54.1%, which is 8.2% higher than that of trRosetta, a mean‐ensemble‐based ResNet model. A head‐to‐head comparison between trRosettaX_FM and trRosetta on the CASP14 targets is shown in Figure 2B. With the exception of 7 targets with slightly lower precision, trRosettaX_FM outperforms trRosetta on 29 targets. We note that significantly higher precision is achieved on the CASP13 dataset than on the CASP14 dataset. The same trend is also observed for other methods participating in both CASP13 and CASP14 (Table S1, Supporting Information). This suggests that CASP14's targets are more difficult than CASP13's targets. Nevertheless, Res2Net also shows a similar improvement over ResNet on the CASP13 dataset (the right panel of Figure 2A).

#### 2.2.2 Inclusion of Homologous Templates is Beneficial

Template‐based modeling dominated the field of protein structure prediction for many years until the recent application of deep learning techniques in *de novo* prediction. It is apparent that the combination of both homologous templates and deep learning should yield the most accurate structure prediction. However, it is not a trivial task to automatically incorporate homologous templates into *de novo* methods such as trRosetta. A few studies were done in this direction in the recent work of AlphaFold2 and RaptorX.^[^ ^51^ ^]^ In trRosettaX, multiple homologous templates are combined based an attention‐based module to provide additional features to the Res2Net‐based network to improve the inter‐residue geometry prediction. The subsequent step of structure realization is the same as trRosetta. Note that for each CASP target, we excluded templates that were released after its entry date in CASP in order to mimic the situation of the CASP experiment.

**Figure 3:**
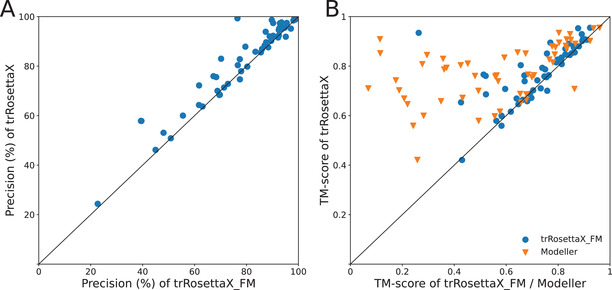
Head‐to‐head comparisons on 54 CASP14 TBM targets. A) Comparison of trRosettaX_FM with trRosettaX based on the precision of the top *L* predicted contacts (sequence separation ≥ 12). B) trRosettaX versus trRosettaX_FM/MODELLER based on TM‐score of the predicted structure models.

**Figure** **3** shows the head‐to‐head comparison between trRosettaX (i.e., with template) and trRosettaX_FM on 54 CASP14 TBM targets. For the top *L* predicted contacts (sequence separation ≥ 12) (Figure 3A), the mean precision of trRosettaX is 0.821 versus 0.788 of trRosettaX_FM. The higher precision results in an improved TM‐score 0.779 by trRosettaX, which is 7% higher than trRosettaX_FM. There are 35 targets with template TM‐score ≥ 0.5, which are detected by HHsearch. On these targets, the average TM‐scores for trRosettaX and trRosettaX_FM are 0.815 and 0.74, respectively. trRosettaX outperforms trRosettaX_FM for all of 35 except only one target with similar accuracy (T1061‐D3, TM‐score is 0.688 vs 0.691). The remaining 19 targets with template TM‐score < 0.5 are all TBM‐hard targets, which suggests that HHsearch mainly aims to detect homologous templates for TBM‐easy targets.^[^ ^52^ ^]^ For these TBM‐hard targets, the average TM‐score for trRosettaX is higher than trRosettaX_FM as well (0.714 and 0.7, respectively).

In addition, the predicted structure models by trRosettaX is compared with the models built by MODELLER^[^ ^53^ ^]^ using the HHsearch alignment, which is to check if the templates have been used effectively by the Res2Net‐based network. Figure 3B shows that our method significantly outperforms MODELLER for almost all TBM targets (50 out of 54). Further analysis shows that 53 targets are successfully folded (i.e., TM‐score ≥ 0.5) by trRosettaX, while MODELLER can only fold 32 targets. The only one target (T1030‐D2) without folded models by trRosettaX is a TBM‐hard target; the model generated by trRosettaX has a TM‐score of 0.421 (compared with 0.258 by MODELLER).

Since HHsearch does not work well for remote homologs, we further compare with MODELLER using the “best” templates obtained by structure alignment. The experimental structure of each target is searched against the PDB chain database (structures released before May 01, 2020) using the mTM‐align server.^[^ ^54^ ^]^ The top 10 templates (sorted by TM‐score) with TM‐score ≥ 0.5 are regarded as the best templates. The top template is used when no template has TM‐score ≥ 0.5. Figure S4A (Supporting Information) presents a head‐to‐head TM‐score comparison between HHsearch and mTM‐align. Using the mTM‐align templates significantly improves the prediction accuracy for almost all TBM targets (45 out of 54). Nevertheless, exceptions for three domains were observed, where the HHsearch templates are significantly more accurate than the mTM‐align templates (red points in Figure S4A, Supporting Information). These domains (T1061‐D3, T1070‐D2, and T1091‐D4) are from multi‐domain targets (the numbers of domains for T1061, T1070, and T1091 are 3, 4, and 4, respectively). Thus the degraded performance by mTM‐align for these targets can be explained by the fact that mTM‐align is for global alignment while HHsearch is for local alignment. The head‐to‐head TM‐score comparison between trRosettaX and MODELLER for CASP14's 54 TBM domains is shown in Figure S4B (Supporting Information). The comparison suggests that trRosettaX (with HHsearch templates) significantly outperforms MODELLER for most targets even though the “best” templates are used in MODELLER, which again demonstrates the effectiveness of our strategy of using templates.

**Figure 4:**
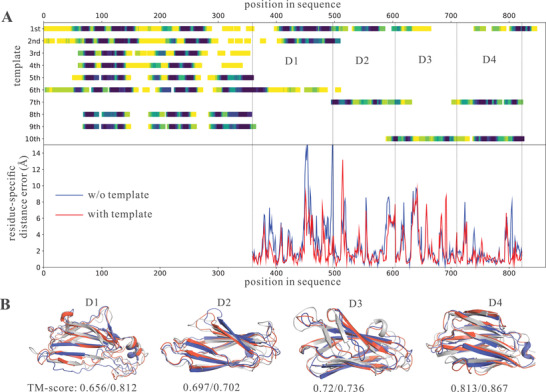
An example to illustrate the improvement due to the inclusion of multiple templates in trRosettaX. This target is T1091 from CASP14. A) Shows the coverage of top 10 templates (the color indicates residue‐specific confidence; darker color indicates higher quality of alignment) and the residue‐specific distance error between the predicted structure models and the experimental structure. B) Is superposition of the first models by trRosettaX_FM (blue) and trRosettaX (red) onto the experimental structure (gray). The numbers below the structures are the TM‐scores of the models predicted by trRosettaX_FM/trRosettaX.

The CASP14 target T1091 is used to illustrate the improvement due to the inclusion of multiple homologous templates in trRosettaX. This target consists of 863 residues and the N‐terminal residues (1‐358) do not have 3D coordinates in the experimental structure. The remaining region consists of four TBM domains (D1‐D4, **Figure** **4**A) according to the official assessment. Figure 4A suggests that no single template can cover all domains. However, when combined the top 10 templates, almost all domains can be covered with confident alignments (marked by dark color in the top half of Figure 4A; obtained from the HHsearch alignment). When no templates are used, trRosettaX_FM can generate models with reasonable accuracy (TM‐score > 0.6 for all domains; see the bottom of Figure 4B). When including these templates in trRosettaX, the models are improved for all domains: D1 and D4 TM‐score > 0.79; D2 and D3 TM‐score > 0.72. The models by both methods are superimposed onto the experimental structure and visualized in Figure 4B, showing that the models by trRosettaX (red cartoon) match better with experimental structure (gray cartoon) than the models by trRosettaX_FM (blue cartoon). The curves in the lower panel of Figure 4A show the residue‐specific distance deviation between the predicted models and the experimental structure, which also suggest that the models generated by trRosettaX (red curve) are mostly more accurate than trRosettaX_FM (blue curve).

### 2.3 Performance in the Blind Test of CASP14

Based on a preliminary version of trRosettaX and trRosettaX_FM, we participated to the blind test of the CASP14 experiment with two groups Yang‐Server and Yang_FM, respectively. Here we present the blind test results of both groups together with the test results after CASP14 of trRosettaX and trRosettaX_FM.

**Figure 5:**
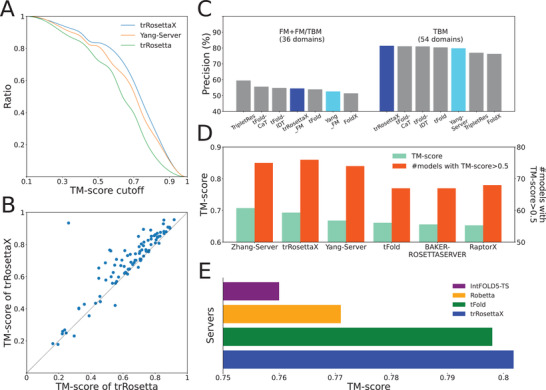
Comparison of the accuracy by different methods on the CASP14 and the CAMEO datasets. A) Comparison between trRosettaX, Yang‐Server and trRosetta on 91 CASP14 targets based on the ratio of targets with TM‐score higher than each TM‐score cutoff. B) Head‐to‐head comparison between trRosettaX and trRosetta based on TM‐score of the predicted models for 91 CASP14 targets. C) The average precision of the top *L* predicted contacts (sequence separation ≥ 12) on CASP14 targets by our methods and other top server groups from CASP14. D) The average TM‐score over CASP14 targets for Yang‐Server, trRosettaX and other representative server groups from CASP14. E) Shows the accuracy of trRosettaX and the top three methods (tFold, Robetta, and IntFOLD5‐TS) on 161 CAMEO targets.

Due to incomplete training and optimization, the tested version in CASP14 is less accurate than the trRosettaX presented in this paper. **Figure** **5**A provides a comparison between trRosettaX, Yang‐Server and trRosetta on 91 targets, where the *x*‐axis represents the TM‐score cutoffs and the *y*‐axis represents the ratio that is defined as the fraction of models with TM‐score greater than certain cutoffs. The closer the curve is to the upper right, the more accurate the corresponding method is. Figure 5A suggests that trRosettaX generates the highest number of models with correct fold, followed by Yang‐Server and trRosetta. The average TM‐score of the models predicted by trRosettaX is 0.693, higher than Yang‐Server (0.668) and trRosetta (0.635). Figure 5B presents a head‐to‐head comparison between trRosettaX and trRosetta on 91 targets, which shows that trRosettaX has higher TM‐score for 80 domains than trRosetta. The head‐to‐head comparisons between trRosettaX and Yang‐Server, trRosetta and Yang‐Server are given in Figure S5 (Supporting Information).

#### 2.3.1 Comparison with Other Contact Prediction Methods

Figure 5C shows the average precision of the top *L* predicted contacts (sequence separation ≥ 12) by our methods and other top server groups in CASP14. For 36 FM targets (T1082 missed for Yang_FM), Yang_FM is ranked at the top 5 (the top 3 after removing method variants from the same lab), with a mean precision 0.526, while trRosettaX_FM rises to the 4th (mean precision 0.542). For 54 TBM targets, Yang‐Server is ranked at the top 4 (the top 2 after removing method variants from the same lab) in all server groups. The improved version of Yang‐Server, i.e., trRosettaX, outperforms all other top groups with a mean precision 0.821.

#### 2.3.2 Comparison with Other Tertiary Structure Prediction Methods

Based on the summed Z‐score (>0.0) over 96 targets, Yang‐Server and Yang_FM are ranked at the 11th and the 13th, respectively, out of 47 structure prediction server groups. After removing method variants from the same lab, Yang‐Server and Yang_FM are at the 5th and the 7th, respectively. Note that Z‐score can only reflect the relative position of a method among others; it does not indicate how accurate the method is. Another assessment based on TM‐score is done by Dr. Zhang (https://zhanglab.ccmb.med.umich.edu/casp14/). According to this assessment, Yang‐Server and Yang_FM are at the 5th and the 6th (2nd and 3rd after removing method variants from the same lab), respectively, over 97 targets (including an additional domain T1048‐D1 which was not evaluated in the official assessment).

Figure 5D and Table S2 (Supporting Information) present the mean TM‐scores of the predicted structure models for 91 CASP14 targets by Yang‐Server, trRosettaX and other representative top servers in CASP14. Yang‐Server/trRosettaX achieves a mean TM‐score of 0.668/0.693, comparable to the top server Zhang‐Server (0.707). Yang‐Server correctly folds the structure with TM‐score over 0.5 for 74 targets, which is close to Zhang‐Server (75). This number is increased to 76 by trRosettaX. Further analysis suggests that trRosettaX has slightly higher TM‐score than Zhang‐Server on 54 TBM targets (0.779 vs 0.776, Figure S6: Supporting Information). Note that the Zhang‐Server's models are based on the comprehensive simulations in I‐TASSER and rich set of templates identified by more than 10 threading methods. In contrast, to speed up, trRosettaX is based on the rapid template search by HHsearch and direct energy minimization with predicted constraints.

Besides Zhang‐Server, it seems that the top ranked server groups are converged with similar methodology. They all make use of deep learning and structure templates but with different approaches. BAKER‐ROSETTASERVER, with a released method named trRosetta2,^[^ ^55^ ^]^ employs deep learning to refine the models generated by trRosetta; while trRosettaX focuses on improving the precision of inter‐residue geometries prediction by re‐designing the deep neural network architecture. We use the multi‐scale network Res2Net, instead of ResNet in trRosetta2. The new network yields more precise geometries prediction, resulting in more accurate structure models. As shown in Tables S2 and S3 (Supporting Information), trRosettaX outperforms BAKER‐ROSETTASERVER significantly at *P*‐value < 0.05 (the average TM‐scores on the CASP14 targets are 0.693 vs 0.656). Especially on FM+FM/TBM targets, the difference is significant at *P*‐value < 10^−4^ (the average TM‐scores are 0.565 vs 0.485).

The method tFold aims to improve the network for distance prediction with a much deeper architecture (≈600 layers). Multiple MSAs are used in both training and inference. In contrast, our method uses Res2Net‐based network with only 50 layers, and only one MSA is fed into the network for each target in both training and inference. Nevertheless, our method outperforms tFold and its variants. For example, on the 91 CASP14 domains, the average precisions of the top *L* contacts (sequence separation ≥ 12) predicted by trRosettaX, tFold, tFold‐CaT, tFold‐IDT are 0.718, 0.698, 0.709, 0.705, respectively; and the average TM‐scores of the predicted structure models are 0.693, 0.661, 0.655, 0.660, respectively.

RaptorX uses deep learning in both threading and *de novo* prediction.^[^ ^51^ ^]^ It focuses on developing deep‐learning‐based threading method and uses ResNet‐based network to predict inter‐residue distance; while trRosettaX feeds the HHsearch templates into Res2Net‐based distance prediction network as extra features. Overall, trRosettaX outperforms RaptorX significantly at the significance level of 0.05 (Tables S2 and S3, Supporting Information): the average TM‐scores are 0.565 versus 0.474 on CASP14's FM+FM/TBM targets; and 0.779 versus 0.775 on the TBM targets.

**Figure 6:**
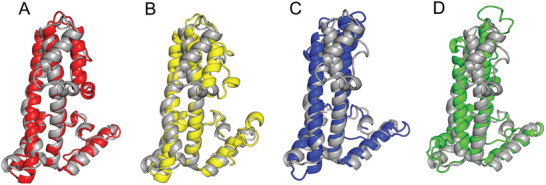
A representative example T1099‐D1 to illustrate the improvement of trRosettaX. A–D) are the superpositions of the models generated by trRosettaX (red), FALCON‐TBM (yellow), Zhang‐Server (blue) and Yang‐Server (green) against the experimental structure (gray).

Table 1 lists a few targets that trRosettaX generates the most accurate structure models among all server groups. We use a representative example T1099‐D1 to illustrate the improvement of our method. The superposition of the models by all methods onto the experimental structure is given in **Figure** **6**. T1099 is a single‐domain protein with 262 residues and classified as a TBM‐hard target, for which 8 homologous templates were detected by HHsearch. The TM‐score of the modeled structure by trRosettaX is 0.76, while the TM‐scores for Yang‐Server and Zhang‐Server are 0.52 and 0.514, respectively. The model with the highest TM‐score in CASP14 was generated by the group FALCON‐TBM (0.647).^[^ ^56^ ^]^ We checked Yang‐Server's prediction log in CASP14 and found that only the top template was used. In contrast, all the 8 templates were used in trRosettaX. This helps improve the model quality significantly from 0.52 to 0.76. Note this target is a *duck hepatitis B virus capsid* possessing an icosahedral structure, which resembles the template structures (PDB IDs 3J2V, 6HU7, 5GMZ, 5T2P, 6UI7, 6ECS, and 6TIK). However, it has distinct features with these templates as its capsid protein has ≈260 rather than ≈180 amino acids in others. Due to such difference, combining multiple templates to cover more regions in the target seems to be important to improve the modeling accuracy. The other three cases listed in Table 1 also support similar conclusions. We also compared the standalone versions of AlphaFold2 and trRosettaX without templates on the targets listed in Table 1. Table S4 (Supporting Information) shows that though less accurate than AlphaFold2, trRosettaX takes much less computer resource and time.

**Table 1:** Four CASP14 targets that trRosettaX builds the most accurate structure models among all server groups. The TM‐scores by trRosettaX, Yang‐Server, Zhang‐Server and the best performing servers are compared

Target ID	trRosettaX	Yang‐Server	Zhang‐Server	Best server in CASP14
T1099‐D1	0.76	0.52	0.514	0.647 (FALCON‐TBM^[^ ^56^ ^]^)
T1094‐D1	0.739	0.608	0.644	0.68 (Zhang‐CEthreader^[^ ^57^ ^]^)
T1060s2‐D1	0.787	0.749	0.77	0.775 (Zhang‐TBM^[^ ^58^ ^]^)
T1101‐D2	0.895	0.81	0.813	0.838 (RaptorX^[^ ^51^ ^]^)

#### 2.3.3 Impact of Training Strategy and MSA Depth

We compare with other top server groups in CASP14 based on target length. As shown in Figure S8 (Supporting Information), with the increase of protein sequence length, our methods maintain good performance on both FM+FM/TBM targets and TBM targets. In other words, our random binary‐sub‐sampling strategy to reduce computer memory during training (see Experimental Section) can effectively address the problem of structure prediction for large proteins.

We evaluate the predictive performance of splitting sequences based on their domain boundaries during training. It can be seen from Table S5 (Supporting Information), using domains, rather than a random selection of sub‐sequences, does not improve the predictive performance. This illustrates that the domain‐selection training may reduce the diversity of training samples; while random sampling of sub‐sequences is appropriate to capture both the intra‐ and inter‐domain interactions.

As MSAs are used in our method, we compare the model quality with the MSA depth on the CASP14 dataset. On the FM+FM/TBM and the TBM targets, the Pearson's correlation coefficients between the TM‐scores of predicted models and the MSA depths are 0.331 and 0.278, respectively (Figure S9, Supporting Information). This suggests that the model quality is only weakly correlated with the MSA depth.

#### 2.3.4 Performance on CAMEO Test Set

We further test trRosettaX on 161 targets from CAMEO over 3 months between June 13, 2020 and September 5, 2020. The accuracy of trRosettaX and the top three methods (tFold,^[^ ^37^ ^]^ Robetta,^[^ ^59^ ^]^ and IntFOLD5‐TS^[^ ^60^ ^]^) is shown in Figure 5E. trRosettaX achieves the best performance with a slightly higher TM‐score (0.802) than tFold (0.798), followed by Robetta (0.771) and IntFOLD5‐TS (0.76). For the 161 targets, 147 targets are foldable (TM‐score > 0.5) by trRosettaX; while tFold, Robetta and IntFOLD5‐TS can fold 146, 143, and 140 targets, respectively. The head‐to‐head comparisons between trRosettaX and other methods are given in Figure S7 (Supporting Information). The red dots indicate that only trRosettaX can fold the targets with TM‐score higher than 0.5 while the green dots are the opposite. We find that all targets marked by green color are very close to the diagonal (indicating similar accuracy for both methods); while the most of the red points are far away from the diagonal. These data demonstrate once again that trRosettaX is robust and accurate.

## 3 Conclusions

We have presented the trRosettaX pipeline, an improved version of the original trRosetta for protein structure prediction by using a new multi‐scale network and an attention‐based module to exploit multiple templates into the network. trRosettaX shows consistent improvement over trRosetta and is competitive with other state‐of‐the‐art automated methods in the CASP and CAMEO experiments.

In spite of this advance, we admit that there is still a significant margin with AlphaFold2 in terms of modeling accuracy. One of the immediate ways to fill the margin is to borrow its idea and improve upon it. Nevertheless, trRosettaX does have a few advantages over AlphaFold2. The first is that trRosettaX is easy to use, which is much faster and takes significantly less computing resource than AlphaFold2. The second is all source codes and data used in trRosettaX are released to the public to speed up the development of more new methods. To the best of our knowledge, the training codes and training data are not released in most of other deep learning‐powered structure prediction methods, including AlphaFold2.

Finally, we observed that both AlphaFold2 and trRosettaX do not perform well on the CASP14 targets for single‐sequence input. In our test, AlphaFold2's average TM‐score for the CASP14 domains drops to 0.34, which is only slightly higher than trRosettaX (0.3). This suggests that both methods rely on the existence of homologous sequences. Thus, MSA‐free modelling maybe one of the directions for future development.

## 4 Experimental Section

### Network Architecture

As shown in Figure 1C, two deep‐learning‐based inter‐residue geometries predictors are developed, one for *de novo* prediction (denoted by Res2Net_FM) and the other for template‐based prediction (denoted by Res2Net_TBM). The input tensor of Res2Net_FM has 526 feature channels, including sequence one‐hot code, position‐specific scoring matrix (PSSM), positional entropy and coevolutionary information (see trRosetta for detail).^[^ ^35^ ^]^ Additional template features are fed into Res2Net_TBM together with the input of Res2Net_FM. The outputs of both networks are then combined based on template qualities and inter‐template weight distribution.

### Res2Net

Different from existing ResNet‐based methods,^[^ ^33^, ^34^, ^45^, ^61^ ^]^ the recently proposed network architecture Res2Net is used,^[^ ^46^ ^]^ designed to obtain multi‐scale features more efficiently, as the network architecture. As illustrated in Figure S3 (Supporting Information) and Figure 1B, unlike the basic ResNet bottleneck block (Figure S3A, Supporting Information), in a Res2Net block (Figure 1B and Figure S3B: Supporting Information), after a 1 × 1 convolution layer, the feature maps, denoted by *x*, are split into several subsets in channel wise, followed by different operations, and fed into another 1 × 1 convolution layer after merge. In detail, if the feature maps *x* is split into subsets *x*_1_, *x*_2_, …, *x_s_* (*s* = 4 in this work), where each *x_i_* walks through its exclusive 3 × 3 convolution layer, denoted by *K_i_*; and the corresponding output *y_i_* can be written as:

(1) $$y_{i}=\left\{\begin{array}{c} x_{i},\;\;i=1\\ K_{i}\left(x_{i}\right),\;\;i=2\\ K_{i}\left(x_{i}+y_{i-1}\right),\;\;2<i\leq s \end{array}\right.$$

### Res2Net_FM

To use Res2Net in the inter‐residue geometries prediction, down‐sampling operations (e.g., pooling and multi‐step convolution) are removed in Res2Net just like the previous ways^[^ ^61^ ^]^ of modifying ResNet. Unlike other ResNet‐based methods consisting of the blocks with two 3 × 3 convolution layers, bottleneck blocks are used as shown in Figure 1B and Figure S3B (Supporting Information), which can make the network deeper and wider without increase of parameters. Each convolution layer is followed by the instance normalization and the ELU activation function.

Specifically, Res2Net_FM (Figure 1C) takes the features derived from a multiple sequence alignment (MSA) as the only input, and the network consists of four groups of Res2Net blocks. The number of filters used in a single convolution layer ranges from 64 to 512; while the number of feature subsets split is fixed to 4 in all Res2Net blocks. After the last Res2Net block, the output tensor is fed into 4 different branches for the 4 prediction tasks (1 distance and 3 orientations). Each branch contains a single 1 × 1 convolution layer, followed by a softmax layer to produce a probability distribution. The input features and label binning method are the same as those in trRosetta.

### Template Selection

In this work, HHsearch is used to detect homologous templates quickly. A template is defined as *good* if its probability (obtained from the HHsearch output, indicating the probability for the query and template HMMs to be homologous) is greater than 60% or its E‐value is less than 0.001. The final geometry prediction for each target is based on the combination of the predicted geometries by Res2Net_FM and Res2Net_TBM, if *good* templates are found. Otherwise, only Res2Net_FM is used.

### Res2Net_TBM

The architecture of Res2Net_TBM is similar to Res2Net_FM, except that the input feature maps of Res2Net_TBM consist of features extracted from templates as well as the coevolution information transformed by the first two Res2Net groups of Res2Net_FM (Figure 1C). In Res2Net_TBM, the utilization of templates are optimized in two aspects. 1) The well‐trained Res2Net_FM network is used as a pre‐trained network to guide the training of TBM networks (the middle arrow of Figure 1C). 2) To obtain as wide‐covered templates information as possible, multiple templates are fed into the Res2Net_TBM network. The feature maps are combined from different templates based on the axial attention^[^ ^62^ ^]^ between coevolution information and templates features (Figure S1, Supporting Information).

### Combining Multiple Templates Using an Attention Module

Figure S1 (Supporting Information) illustrates how to combine the features from multiple templates in Res2Net_TBM. For each target, after obtaining the top *N* templates by HHsearch (*N* ≤ 10 here), the inter‐residue C_*β*_‐C_*β*_ distance and three kinds of orientations for each template is first calculated. They are converted into bins as in trRosetta followed by one‐hot encoding. In total, this produces 100 ( = 37 for *d* + 25 for *ω* + 25 for *θ* + 13 for *φ*) feature maps (with shape of *L* × *L* × 100, *L* is the length of query sequence) for each template. The *N* groups of feature maps are then fed into axial attention module as a batch of samples (i.e., tensors of dimension *N* × *L* × *L* × 100) along with MSA features to generate the spatial attention maps, which contain the pixel‐wise attention values for each template. A softmax layer follows to transform the attention values into pair‐wise weights for each template. The final feature maps from combined templates are calculated by the pair‐wise weighted summation of the features from all templates. Both the softmax and the sum operations are carried out along the *batch dimension*.

### Incorporating Template Information with MSA Information by Pre‐Trained Method

As shown in Figure 1C, for Res2Net_TBM, instead of using template information only, the features derived from MSA are reused and fed into the well‐trained Res2Net_FM network. The output feature maps of the second Res2Net group are then concatenated with the feature maps from the templates, followed by 4 newly‐defined Res2Net groups and the final classifier. Therefore, Res2Net_TBM contains 6 groups of Res2Net blocks: the first two are from the pre‐trained FM network and the remaining four are newly constructed. Only the parameters in the last four Res2Net groups are updated during training.

### Combination of the Predictions from Res2Net_FM and Res2Net_TBM

As mentioned above, the final inter‐residue geometries prediction is the combination of the outputs from Res2Net_FM and Res2Net_TBM. First, for the *k* ^th^ template, the 2‐site residue‐specific confidence scores are calculated, *s*_k_(*i*, *j*) = [*s*_k_(*i*)+*s*_k_(*j*)]/2, where *s_k_* (*i*) (in [0,1]) is the *k* ^th^ template's confidence score at the *i* ^th^ residue (from the HHsearch output). Then, the weight for the TBM‐based prediction is estimated as$w_{TBM}\left(i,j\right)=\sum_{k=1}^{N}w_{k}\left(i,j\right)s_{k}\left(i,j\right)$, where *w_k_* (*i*, *j*) is obtained from the axial attention module. Note that if no *good* templates are detected, *w*_TBM_ is set to 0 for all residue pairs. The weight for the prediction from Res2Net_FM is *w_FM_* (*i*,*j*) = 1 − *w_TBM_* (*i*,*j*). For each residue pair in the query sequence, the final predicted geometries are the weighted average of the predictions by Res2Net_FM and Res2Net_TBM using the above weights.

### Datasets

The training set of the method is the same as that used in trRosetta, containing 15 051 non‐redundant (< 30% pairwise sequence identity) protein chains (available for download at the website https://yanglab.nankai.edu.cn/trRosetta/benchmark/). The proportion of training/validation split is 95/5%, which means 14 299 chains are used for training and other 752 for validation.

Three independent test sets are used to analyze and compare the methods. The first set is 91 domains from CASP14, including 37 FM+FM/TBM and 54 TBM domains, after excluding two targets (T1085 and T1086, involving 6 domains), for which there is no experimental structures. The second set contains 31 FM domains from CASP13, which was used in trRosetta. The third set contains 161 targets from the CAMEO experiments between June 13, 2020 and September 5, 2020. All predictions by the methods are based on the full‐length sequences without domain parsing. PDB templates released after the target date in the corresponding prediction season are excluded in all experiments.

### Training

The MSAs used in trRosettaX are the same as that used in trRosetta. In addition to the training strategies used in trRosetta, such as MSA subsampling, a special data augmentation method is implemented for big samples that are too long to be fed into the network. Due to the limited GPU memory (11–12G), only samples with less than 260 residues can be accepted in the network. In trRosetta, a continuous sub‐sequence is simply sampled randomly and then fed it into the network, which may lead to loss of information for big proteins (see Figure S2A, Supporting Information). In contrast, in this work, for large targets with more than 260 residues, in addition to the sub‐sequences produced from single‐sub‐sampling, two sub‐sequences, one from the first half of the original sequence and another from the second half are randomly sampled, and then stitch them together to form a new sequence (Figure S2B, Supporting Information). This sub‐sampling is repeated in every epoch so that the generated sequences are distinct, serving as one aspect of data augmentation.

### Folding by Energy Minimization

The structure prediction procedure is the same as that in trRosetta. Briefly, a two‐step approach is adopted. The predicted distance and orientations are first converted into energy functions. A set of 120 coarse‐grained centroid models are generated based on energy minimization in PyRosetta.^[^ ^63^ ^]^ The top five centroid models with the lowest energy are then submitted to the second step of full‐atom relaxation. The model with the lowest full‐atom energy is selected as the final model.

### Statistical Analysis

The significance level of the TM‐score difference between the method and others is obtained based on statistical tests. Half of the proteins are randomly selected from the test set and then calculate the average TM‐score for each pair of methods. This experiment is repeated 10 times, producing 10 paired samples. The Anderson‐Darling test is first used to test if the samples are from a normal distribution. If so, the two‐tailed paired Student's *t*‐test is applied to examine the statistical significance. Otherwise, the nonparametric Wilcoxon signed‐rank test is utilized. *P*‐values < 0.05 are considered to be statistically significant. The statistical analysis is done using the Python libraries *NumPy* ^[^ ^64^ ^]^ and *SciPy*.^[^ ^65^ ^]^

## Conflict of Interest

The authors declare no conflict of interest.

## Supporting information

Supporting Information

## Data Availability

https://yanglab.nankai.edu.cn/trRosetta/benchmark/
