# Supplementary material for: Improved Protein Structure Prediction Using a New Multi‐Scale Network and Homologous Templates
Source: Adv Sci (Weinh). 2021 Oct 31;8(24):2102592. doi: 10.1002/advs.202102592 (PMC8693034; doi:10.1002/advs.202102592)
Supplement: Supplementary file 1 — Supporting Information [file ADVS-8-2102592-s001.pdf]

Supplementary Material

Table S1. The precision of the top *L* predicted contacts (sequence separation ≥ 12) by methods that participate to both CASP13 and CASP14. The results were from the respective CASP websites.

Methods	CASP14<br>(23 FM+14 FM/TBM targets)	CASP13<br>(31 FM targets)
RaptorX-contact	0.484	0.602
TripletRes	0.592	0.598
Yang-Server	0.517	0.551
ZHOU-contact	0.411	0.511

Table S2. Comparison of TM-scores between trRosettaX and other top methods in CASP14.

Dataset	Zhang-Server	trRosettaX	Yang-Server	tFold	BAKER-ROS.	RaptorX
FM+FM/TBM (37)	0.606	0.565	0.53	0.525	0.485	0.474
TBM (54)	0.776	0.779	0.763	0.758	0.771	0.775
All (91)	0.707	0.693	0.668	0.661	0.656	0.653

Table S3. The *p*-values of the statistical tests between trRosettaX and other top server groups in CASP14 based on the TM-scores presented in Table S2. (-) indicates that trRosettaX has a lower TM-score than the compared method.

Dataset	Zhang-Server	Yang-Server	tFold	BAKER-ROS.	RaptorX
FM+FM/TBM (37)	0.0001(-)	$7.1*10^{-5}$	0.001	$3.0*10^{-5}$	$3.9*10^{-7}$
TBM (54)	0.0420	0.0001	0.0002	0.0329	0.0236
All (91)	0.0238(-)	$6.5*10^{-6}$	$3.9*10^{-5}$	0.0002	$1.1*10^{-5}$

Table S4. Comparison between trRosettaX_FM and AlphaFold2 on representative domains listed in Table 1. The comparisons are shown in "trRosettaX_FM/AlphaFold2" format. All experiments were performed on our Linux server with 24 CPU cores and 128 GB memory. We directly fed the prepared MSA into trRosettaX_FM and AlphaFold2 rather than searching sequence databases during inference. The prediction results of AlphaFold2 were obtained through running its open-source codes (using model_1 only) with the same MSA used for trRosettaX_FM and no template was used. Note that trRosettaX_FM utilized only one CPU core, which is more friendly for common personal computers, while AlphaFold2 used all CPU cores (24 in our system) during inference.

Target ID	Length	CPU cores	Time (min.)	Memory (GB)	TM-score
T1099-D1	262	1/24	21/56	1.65/4.14	0.534/0.857
T1094-D1	496	1/24	106/133	2.82/5.91	0.744/0.921
T1060s2-D1	298	1/24	29/74	1.83/4.51	0.731/0.914
T1101-D2	318	1/24	34/72	1.88/4.58	0.768/0.939

Table S5. Comparison of the precision of the top *L* predicted contacts (sequence separation ≥ 12) with models trained based on domain splits. Note that single model is used here.

Methods	CASP14 (23 FM+14 FM/TBM targets)	CASP13 (31 FM targets)
Random split	0.650	0.724
Domain-based split	0.648	0.723

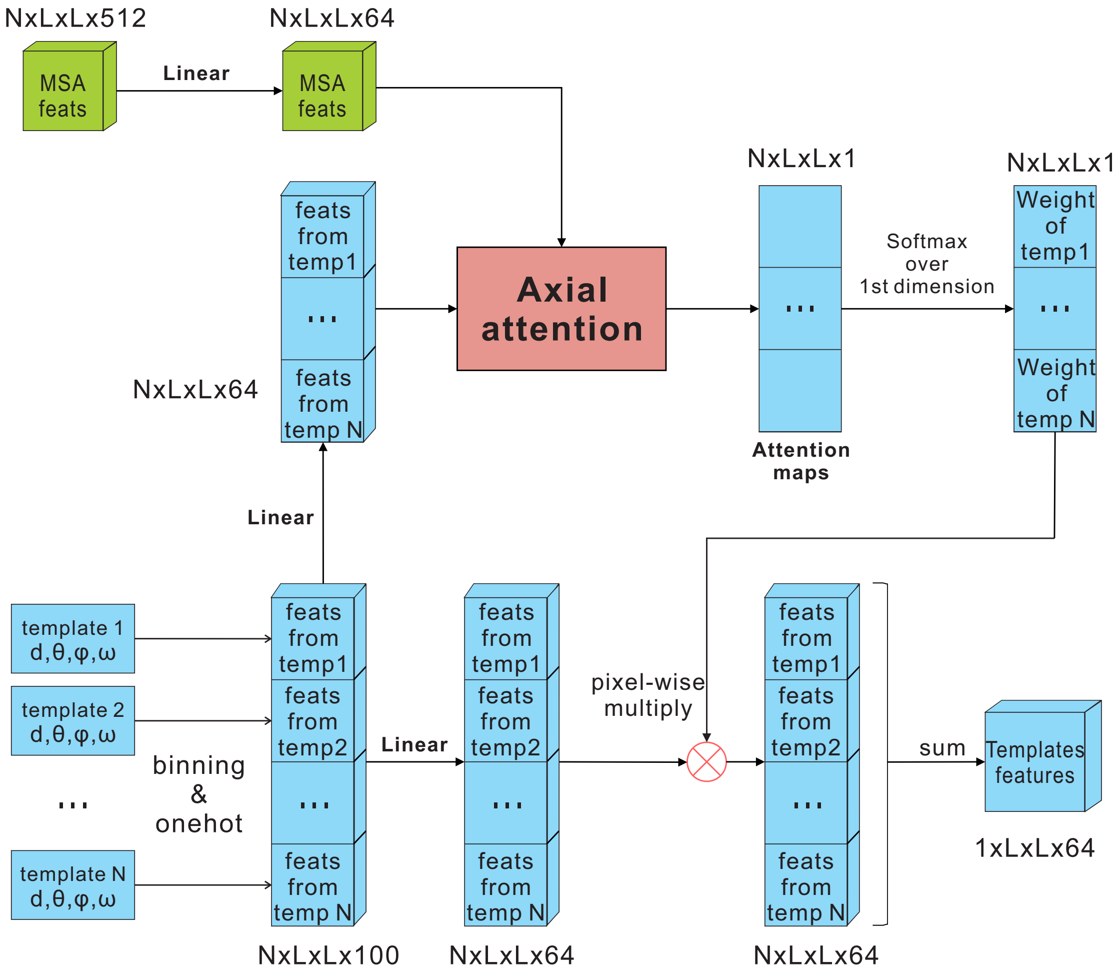

Figure S1. Combining information from multiple templates with an axial attention module. From the top N templates, we first calculate inter-residue geometries and preprocess them into one-hot encodes, followed by concatenation over the first dimension, producing feature maps with the shape $N \times L \times L \times 100$. Then we calculate the axial attention maps between templates features and MSA features, which are both processed by their respective linear projections. Softmax function is applied to the 1st dimension of the $N \times L \times L \times 1$ attention map to obtain the pixel-wise weights of each template. The weight maps corresponding to different templates are multiplied with original feature maps (processed by a linear projection) and sum together as the final combined feature maps of the top N templates.

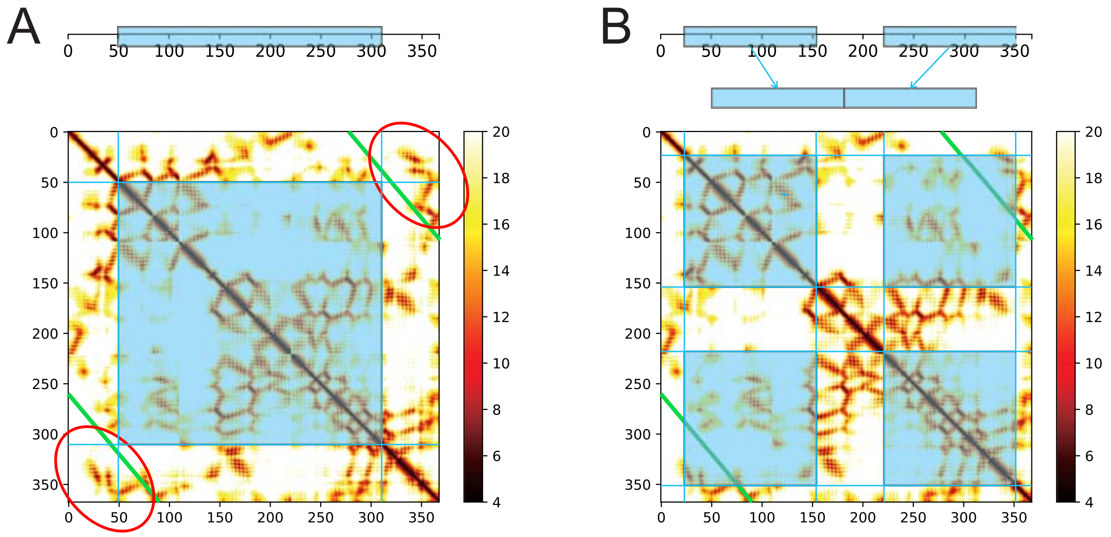

Figure S2. Comparison between single-sub-sampling and binary-sub-sampling. (A) Sampling a continuous single-sub-sequence can lead to the loss of very-long-range information (highlighted by the red circles). (B) By sampling two sub-sequences from both sides of the original sequence, very-long-range information can be captured.

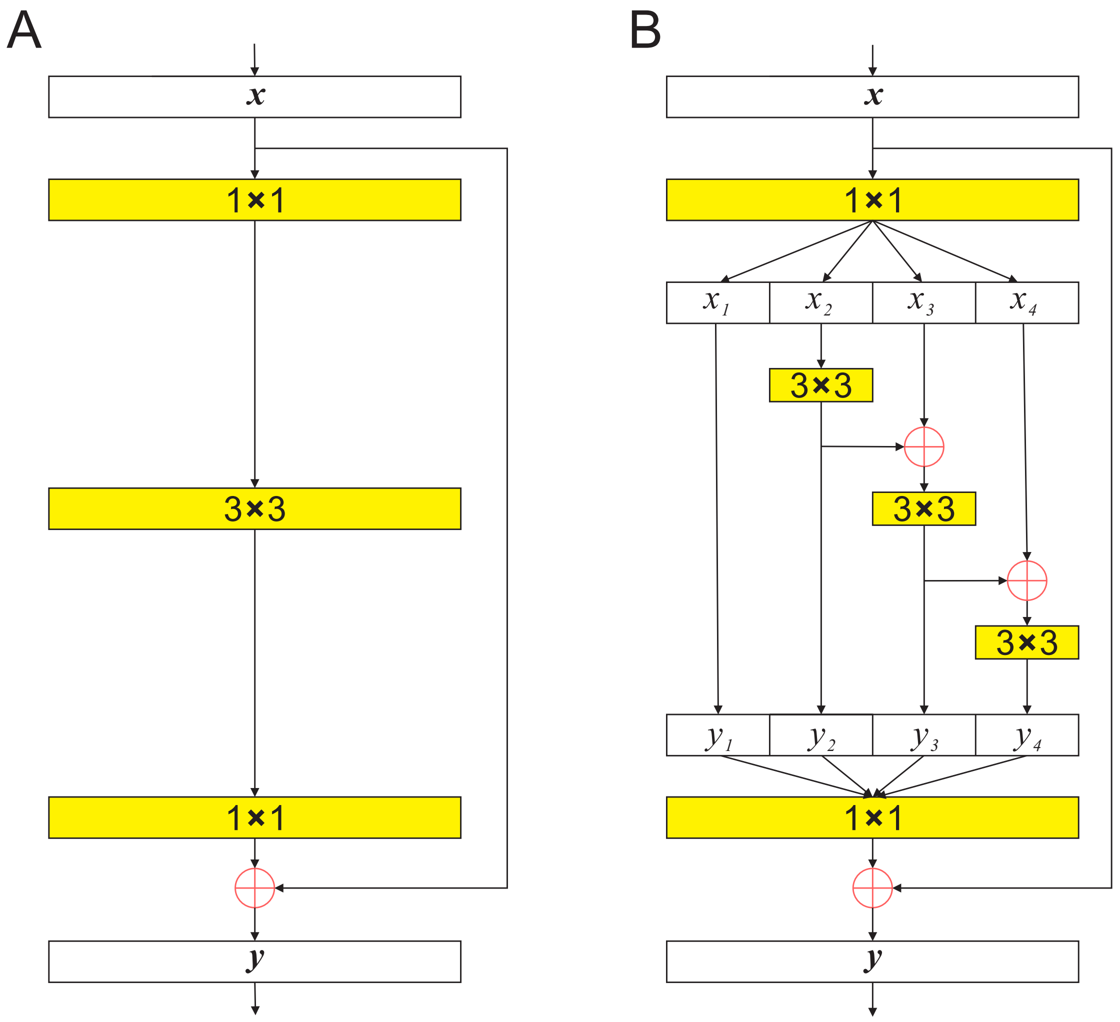

Figure S3. Comparison between a basic ResNet bottleneck block (A) and a typical Res2Net block (B). Here we use *receptive field*, reflecting how large range of input information can be covered in a single pixel point of output, to illustrate how Res2Net obtains more efficient multi-scale features. The receptive field sizes of y_1, y_2, y_3, y_4 are 1, 3, 5 and 7, respectively. Thus, the output of a Res2Net block can mix feature maps extracted by operations that achieve different receptive field sizes. In contrast, the receptive field sizes are 3 in all feature maps handled by a ResNet block.

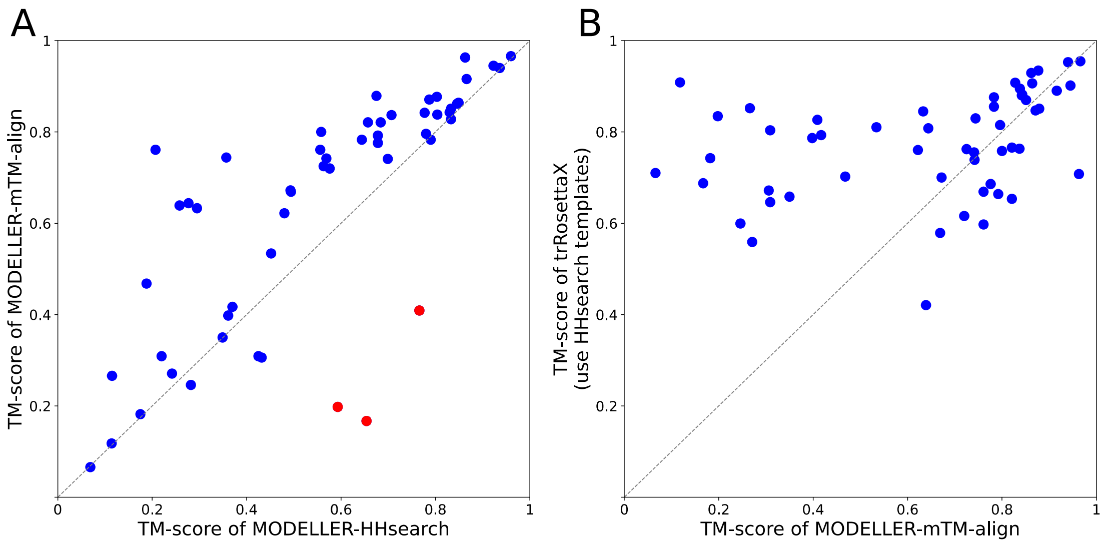

Figure S4. Head-to-head comparisons on 54 CASP14 targets. (A) MODELLER using templates from HHsearch (denoted by MODELLER-HHsearch) vs using best templates detected by mTM-align (denoted by MODELLER-mTM-align). (B) trRosettaX (with HHsearch templates) vs MODELLER-mTM-align.

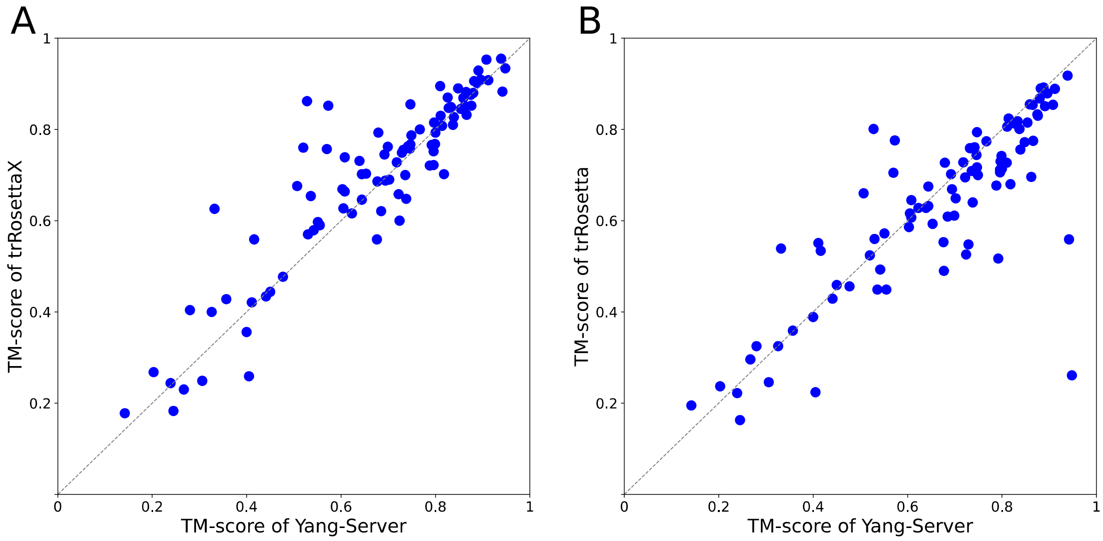

Figure S5. Head-to-head comparisons on 91 CASP14 targets. (A) trRosettaX vs Yang-Server. (B) trRosetta vs Yang-Server.

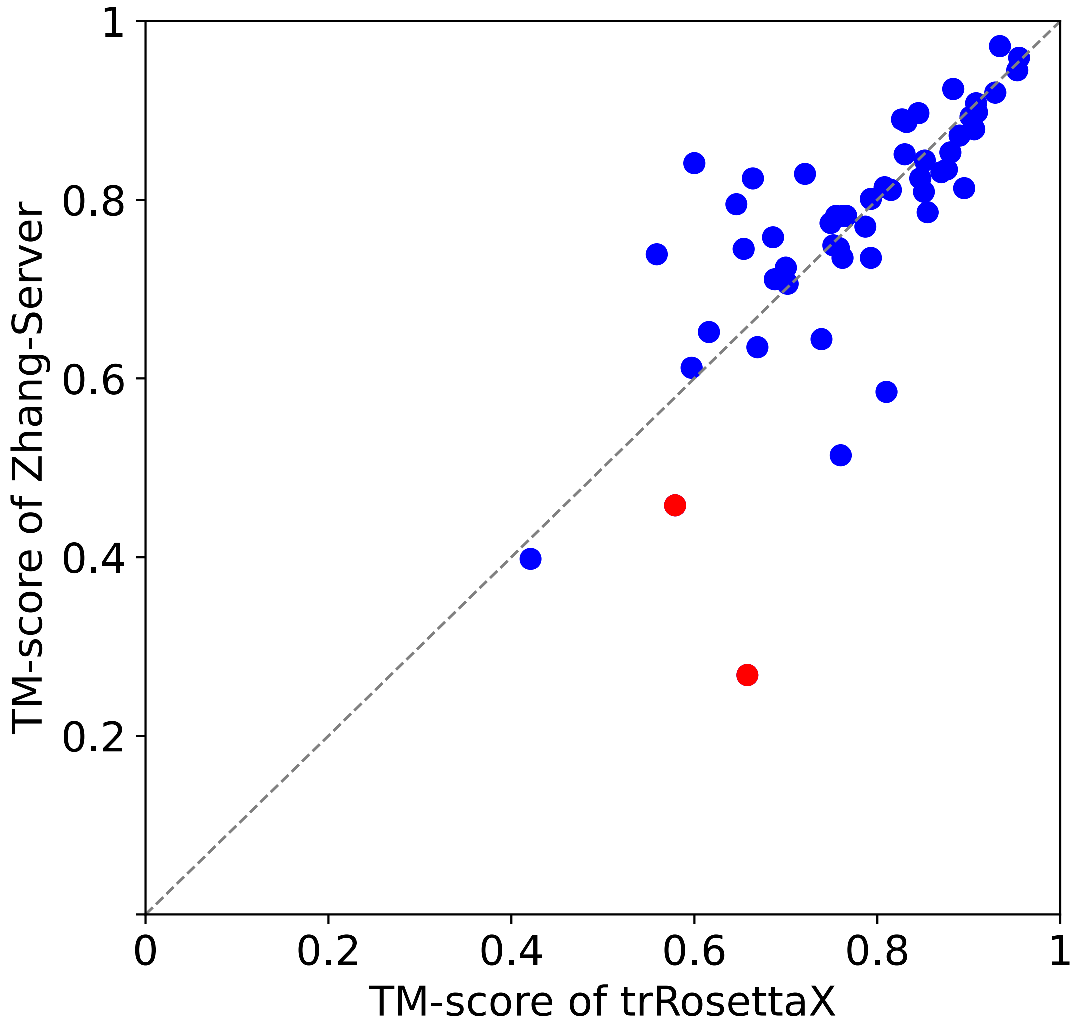

Figure S6. Head-to-head comparisons between Zhang-Server and trRosettaX on 54 TBM targets. Red points indicate that targets are foldable (i.e., TM-score > 0.5) by trRosettaX only.

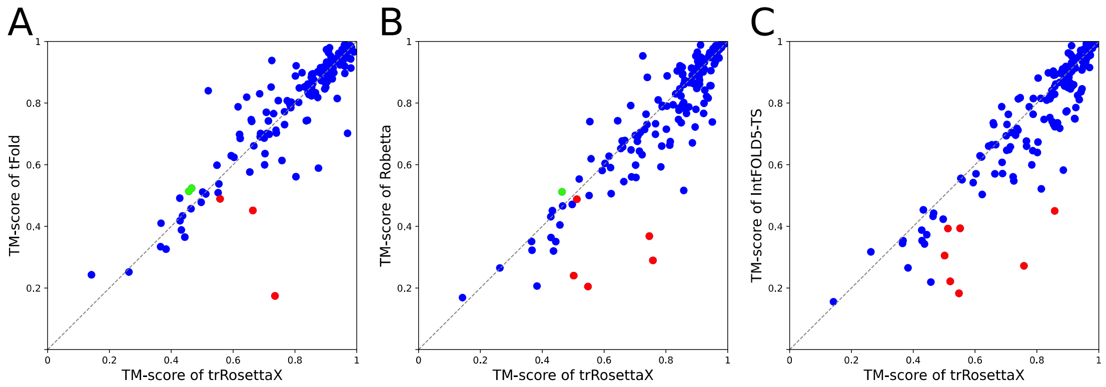

Figure S7. Head-to-head comparisons on 161 targets from CAMEO experiment. (A, B, C) trRosettaX vs tFold, Robetta and IntFOLD5-TS, respectively. The red dots indicate that only trRosettaX can fold the targets with TM-score higher than 0.5 while the green dots are the opposite.

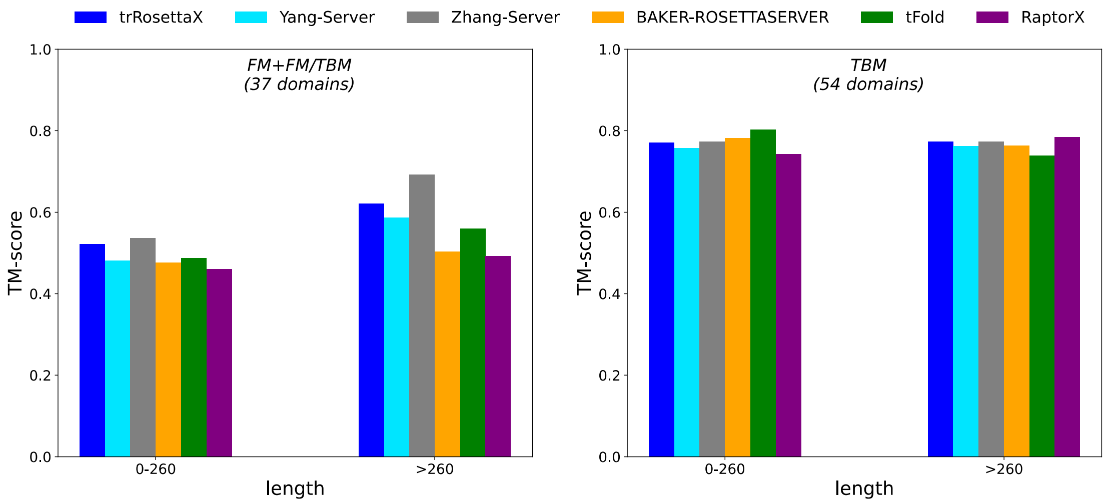

Figure S8. Comparison with other top servers on targets with different lengths.

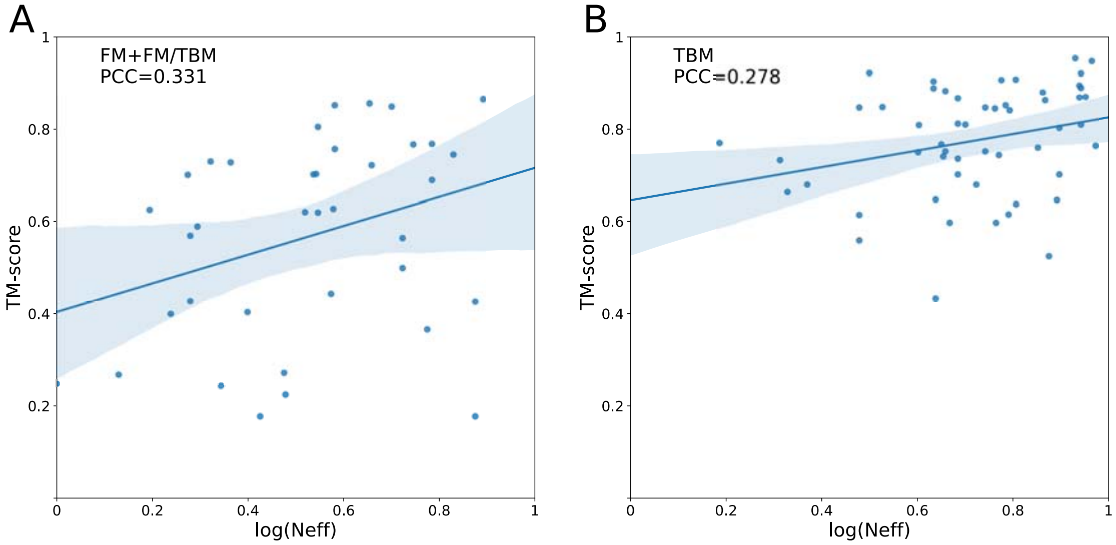

Figure S9. Pearson's correlation coefficient (PCC) between trRosettaX's model quality (TM-score) and the logarithm of the MSA depth on the CASP14 targets. The depth is measured by the effective number of homologous sequences in MSA at 80% sequence identity. (A) 37 FM+FM/TBM targets. (B) 54 TBM targets.
